# Prognostic factors affecting survival in patients with non-small cell lung cancer treated with salvage surgery after drug therapy: a multi-institutional retrospective study

**DOI:** 10.1186/s12957-023-03177-5

**Published:** 2023-09-15

**Authors:** Shigeki Suzuki, Keisuke Asakura, Masayuki Okui, Naoko Izawa, Makoto Sawafuji, Hiroyuki Sakamaki, Takao Shigenobu, Atsushi Tajima, Naoyuki Oka, Kyohei Masai, Kaoru Kaseda, Tomoyuki Hishida, Hiroyuki Yasuda, Koichi Fukunaga, Hisao Asamura

**Affiliations:** 1 Division of Thoracic Surgery, Department of Surgery, Keio University School of Medicine, 35, Shinanomachi, Shinjyuku-ku, Tokyo 160-8582 Japan; 2 Department of General Thoracic Surgery, Sagamihara Kyodo Hospital, Sagamihara, Kanagawa Japan; 3 Department of General Thoracic Surgery, Kawasaki Municipal Hospital, Kawasaki, Kanagawa Japan; 4 Department of General Thoracic Surgery, Saiseikai Utsunomiya Hospital, Utsunomiya, Tochigi Japan; 5 Division of Pulmonary Medicine, Department of Medicine, Keio University School of Medicine, Shinanomachi, Shinjyuku-ku, Tokyo Japan

**Keywords:** Carcinoembryonic antigen, Drug therapy, Non-small cell lung cancer, Salvage surgery

## Abstract

**Background:**

The prevalence of salvage surgeries after drug therapy for non-small cell lung cancer (NSCLC) has risen, mainly due to recent progress in molecular-targeted drugs and immune checkpoint inhibitors for NSCLC. While the safety and effectiveness of salvage surgery after drug therapy for NSCLC have been studied, its indications remain unclear. We aimed to identify the prognostic factors affecting survival in patients with advanced-stage (stages III–IV) NSCLC treated with salvage surgery after drug therapy.

**Methods:**

A retrospective investigation was conducted on patients who received salvage surgery after drug therapy at four hospitals between 2007 and 2020. Salvage surgery was defined as surgery after drug therapy for local progression, tumor conversion to resectable status, and discontinuation of prior drug therapy owing to serious complications.

**Results:**

Thirty-two patients received cytotoxic agents alone (*n* = 12 [38%]), tyrosine kinase inhibitors (TKIs; *n* = 16 [50%]), or immune checkpoint inhibitors (*n* = 4 [13%]) as prior drug therapy. In 11 (34%) and 21 (66%) patients, the clinical stage before treatment was III or IV, respectively. The median initial and preoperative serum carcinoembryonic antigen (CEA) levels were 10.2 (range, 0.5**–**1024) ng/mL and 4.2 (range, 0.6**–**92.5) ng/mL, respectively. Among the patients, 28 (88%) underwent lobectomy, 2 (6%) underwent segmentectomy, and 2 (6%) underwent wedge resection. Complete resection of the primary lesion was accomplished in 28 (88%) patients. Postoperative complications were documented in six (19%) patients. Mortality rates were 0% at 30 days and 3% at 90 days post-operation. The 5-year overall survival rate stood at 66%, while the 5-year progression-free survival rate was 21%. Multivariate analyses showed that prior TKI therapy and preoperative serum CEA level < 5 ng/mL were prognostic factors influencing overall survival (hazard ratio [95% confidence interval]: 0.06 [0.006**–**0.68] and 0.03 [0.002**–**0.41], respectively). The 5-year overall survival in the 11 patients with both favorable prognosticators was 100%.

**Conclusions:**

In this study, prior TKI therapy and preoperative serum CEA level < 5 ng/mL were favorable prognostic factors for overall survival in patients with NSCLC treated with salvage surgery. Patients with these prognostic factors are considered good candidates for salvage surgery after drug therapy.

**Supplementary Information:**

The online version contains supplementary material available at 10.1186/s12957-023-03177-5.

## Background

During the past three decades, salvage surgery for locally recurrent or persistent tumors after radiotherapy (RT) or chemoradiotherapy (CRT) has been performed for many types of malignancies, including head and neck, esophageal, and cervical cancers [1–3]. In 1991, salvage surgery was first introduced in lung cancer as “surgical resection of locally recurrent or persistent tumor after definitive medical treatment” specifically for small cell lung cancer [4]. Since the 2000s, the key focus of salvage surgery for lung cancer has transitioned to non-small cell lung cancer (NSCLC). In the context of NSCLC, the surgical resection of residual or locally recurrent lesions following definitive CRT is generally considered a strict form of salvage surgery [5, 6]. Furthermore, conversion surgery after drug therapy, including the molecular targeted drug, has evolved as a form of broad sense salvage surgery. This procedure is intended for NSCLC that has transitioned from an “originally unresectable” to a “potentially resectable” condition as a result of an excellent response to drug therapy [7].

Recently, molecular-targeted drugs and immune checkpoint inhibitors (ICIs) have significantly enhanced the response rate and the survival of patients with stage IV NSCLC. Consequently, the number of potential candidates for salvage surgery has increased [8]. Although many studies have been conducted on salvage surgery in NSCLC, there are limited studies on surgery after systemic drug therapy, and prognostic factors after salvage surgery following drug therapy are not fully investigated [9]. We have previously reported the safety and effectiveness of salvage surgery for NSCLC after CRT, RT, or drug therapy, but the prognostic factors after drug therapy were not analyzed in that study [10]. Therefore, the current study aimed to identify favorable prognosticators in patients receiving salvage surgery after systemic drug therapy for advanced-stage NSCLC.

## Patients and methods

### Patient selection

This study was a part of the multi-institutional retrospective study “A retrospective study on safety and efficacy of salvage surgery for NSCLC.” This retrospective cohort study was approved by the Ethics Committee of Keio University School of Medicine (approval no. 20200092) and the ethical committees of the other three institutions. Information about the study was communicated via the websites of the four participating institutions, and the requirement for patient consent was waived. Data were retrospectively collected on patients who underwent various salvage surgeries after CRT, RT, and drug therapy with curative intent at Keio University Hospital and its three affiliated institutions from January 2007 to December 2020. We have previously reported on the clinical importance of salvage surgeries in 46 patients with 10 patients undergoing CRT, 4 patients undergoing RT, and 32 patients receiving drug therapy [10]. In this study, we collected further details, including drug therapy regimens and driver mutation status, on 32 of the 46 patients who underwent salvage surgery after drug therapy to clarify the prognostic factors in these patients.

Based on the previous studies [5, 9], we defined salvage surgery after drug therapy as surgery for (1) local tumor progression after prior drug therapy, (2) tumor conversion to resectable status after drug therapy by a restaging of residual tumor, and (3) discontinuation of prior drug therapy due to severe complications. The selection criteria included the following: (1) salvage surgeries defined above, (2) pathological diagnosis of NSCLC at any given time, (3) the absence of a pre-planned surgical procedure, and (4) radiological diagnosis of persistent or locoregionally recurrent tumor. To exclude surgery performed after neoadjuvant chemotherapy, in line with the selection criteria in clinical trials regarding neoadjuvant therapy [11, 12], patients receiving cytotoxic chemotherapy of three courses or fewer courses were excluded.

### Surgery and postoperative follow-up

The methods of postoperative follow-up differ across institutions; however, routine procedures include tumor marker evaluations and chest X-ray examinations every 3 months, along with chest**-**abdominal computed tomography (CT) scans every 6 months. Further assessments, such as brain contrast magnetic resonance imaging and positron emission tomography, are conducted when necessary. In cases when recurrence is suspected, an effort is made to confirm the diagnosis pathologically through biopsy. Postoperative drug therapy is implemented in cases where complete resection is not achieved or when the physician deems it required.

### Clinicopathological characteristics

The patients were categorized into three groups based on the drug type administered before salvage surgery, i.e., cytotoxic agent alone, tyrosine kinase inhibitor (TKI, including TKI use before or after cytotoxic chemotherapy), or ICI (including ICI plus cytotoxic agent). A summary of the clinicopathological characteristics of all patients, as well as the individual groups, have been made. Tumor staging was accomplished using the seventh edition of the tumor**-**node**-**metastasis classification. For the histological assessments, the World Health Organization classification was employed. The Pathological therapeutic effect (Ef) of prior therapy was determined by examining resected specimens (Ef.3, no residual tumor; Ef.2, residual tumor ≤ 1/3 of the lesion; Ef.1, residual tumor ≥ 1/3 of the lesion; Ef.0, no therapeutic effect).

### Evaluation of safety and effectiveness of salvage surgery

To ascertain safety, we analyzed the rate of complications and mortality rates at 30 and 90 days. Furthermore, we examined the duration of the operation, the volume of blood lost, the lengths of chest tube placement, and the length of the postoperative hospitalization. The complications were assessed according to the Common Terminology Criteria for Adverse Events (CTCAE) version 4.0 or Clavien**-**Dindo classification version 2.0 [13]. To assess the efficacy, we calculated 2- and 5-year overall survival (OS) and progression-free survival (PFS) from the date of salvage surgery. We also explored the prognostic determinants for OS and PFS after salvage surgery.

### Statistical analysis

Univariate analysis for clinical characteristics was not conducted because the number of patients in each group was small when divided into groups by prior drug therapy, and there was some overlap in the drugs used. Clinical characteristics are grouped and described as reference only. For survival analysis, the Kaplan**-**Meier method was applied, and the differences between the groups were evaluated using the log-rank test. The Cox regression model was employed in multivariate analysis to detect factors predicting survival. Variables with *p* < 0.15 in univariate analysis were included in multivariate analysis. All calculated *p*-values were two sided, with a threshold of *p* < 0.05 established as indicative of statistical significance. Statistical computations were performed with EZR (Jichi Medical University Saitama Medical Center, Saitama, Japan), a graphical user interface for R (The R Foundation for Statistical Computing, Vienna, Austria).

## Results

### Patient and tumor features

During the past 14 years, out of 4984 patients who received lung cancer surgery, 46 (0.9%) underwent salvage surgery for NSCLC. Among them, 32 (67%) patients underwent salvage surgery after drug therapy. The type of prior drug therapy before salvage surgery was platinum-doublet chemotherapy regimens in 12 (38%) patients (cisplatin regimen, *n* = 8 and carboplatin regimen, *n* = 4), TKI therapy in 16 (50%) patients (epidermal growth factor receptor [EGFR]-TKI, *n* = 15 and anaplastic lymphoma kinase [ALK]-TKI, *n* = 1), and ICI therapy alone or ICI plus cytotoxic chemotherapy in four (13%) patients (pembrolizumab, *n* = 1; pembrolizumab plus platinum-doublet chemotherapy regimens, *n* = 2; and nivolumab plus platinum-doublet chemotherapy regimens, *n* = 1). In the TKI group, nine of 16 patients received TKI therapy alone, whereas the remaining patients received cytotoxic chemotherapy before or after TKI therapy. Patients in the ICI group received a mean of 23 (range, 15**–**35) cycles of ICI therapy. Patients in the cytotoxic agent group received four to six cycles of platinum-doublet chemotherapy; six of 12 patients were treated in combination with bevacizumab. In addition, two patients received second-line chemotherapy with nab-paclitaxel. Furthermore, local therapy for the distant metastasis was performed in six patients with stage IV disease (RT for muscle metastasis in one (3%), RT for bone metastasis in three (9%), surgery for brain metastasis in one (3%), and surgery plus RT for brain metastasis in one (3%) patients). Although we collected information on 32 patients from 2007 to 2020, 14 of 16 (88%) patients who received prior TKI therapy had surgery in the latter half of the study period (2013**–**2020). All four surgeries following ICI therapy were performed in the final 2 years of the study (2019**–**2020). Thus, there was an increase in the number of patients who underwent salvage surgeries after TKI or ICI therapy.

Patient and tumor characteristics according to the type of prior drug therapy are presented in Table 1. In total, 18 (56%) patients were men, and the median patient age at diagnosis was 63.5 (range, 41**–**80) years. Furthermore, 31 (97%) patients had an *ECOG performance status (PS)* of 0, and 17 (53%) patients had some form of comorbidity. The pre-treatment clinical stage was IIIA, IIIB, and IV in four (13%), seven (22%), and 21 (66%) patients, respectively. The posttreatment clinical (yc) stage was stages I, II, IIIA, IIIB, and IV in 16 (50%), seven (22%), four (13%), four (13%), and one (3%) patient, respectively. The median initial serum carcinoembryonic antigen (CEA) level was 10.2 (range, 0.5**–**1024) ng/mL, while the preoperative (just before surgery) serum CEA level (hereinafter called “preoperative CEA”) was 4.2 (range, 0.6**–**92.5) ng/mL. Preoperative CEA was not measured in two patients. The initial and preoperative CEA level was 17.1 (range, 0.8**–**381) ng/mL and 3.7 (range, 0.6**–**73) ng/mL in the TKI group, 6.8 (range, 0.5**–**1024) ng/mL and 6.2 (range, 1.4**–**92.5) ng/mL in the cytotoxic agent group, and 7.4 (range, 5.4**-**15.1) ng/mL and 5 (range, 3.4**–**5.1) ng/mL in the ICI group, respectively. The difference in initial and preoperative CEA levels was the largest in the TKI group.

**Table 1:** Patient and tumor characteristics according to the type of prior drug therapy

Characteristics	Cytotoxic agent (*n* = 12)	TKI (*n* = 16)	ICI (*n* = 4)	All drugs (*n* = 32)
Median age at diagnosis, years (range)	62.5 (52–77)	62.5 (41–80)	71.5 (62–75)	63.5 (41–80)
Male sex	9 (75)	5 (31)	4 (100)	18 (56)
Median smoking index (range)	935 (0–1400)	200 (0–3000)	925 (700–1200)	750 (0–3000)
ECOG performance status				
0	11 (92)	16 (100)	4 (100)	31 (97)
1	1 (8)	0	0	1 (3)
Comorbidities				
COPD	2 (17)	1 (6)	3 (75)	6 (19)
Interstitial pneumonia	2 (17)	0	0	2 (6)
Diabetes mellitus	1 (8)	2 (13)	0	3 (9)
Cardiac disease	4 (33)	1 (6)	0	5 (16)
Cerebrovascular disease	1 (8)	0	0	1 (3)
Initial serum CEA level, ng/mL (range)	6.8 (0.5–1024)	17.1 (0.8–381)	7.4 (5.4–15.1)	10.2 (0.5–1024)
Preoperative serum CEA level, ng/mL (range)	6.2 (1.4–92.5)	3.7 (0.6–73)	5 (3.4–5.1)	4.2 (0.6–92.5)
Clinical nodal metastasis (+)	8 (67)	10 (63)	3 (75)	21 (66)
Pre-treatment clinical stage (c stage)				
IIIA	3 (25)	0	1 (25)	4 (13)
IIIB	3 (25)	2 (13)	2 (50)	7 (22)
IV	6 (50)	14 (88)	1 (25)	21 (66)
Initial distant metastatic organ*				
Brain	2 (17)	0	0	2 (6)
Bone	2 (17)	6 (38)	0	8 (25)
Lung	0	3 (19)	0	3 (9)
Adrenal gland	0	1 (6)	0	1 (3)
Pleura	1 (8)	5 (31)	1 (25)	7 (22)
Muscle	1 (8)	0	0	1 (3)
Others	1 (8)	1 (6)	0	2 (6)
Number of distant metastatic organs				
1	5 (42)	10 (63)	1 (25)	16 (50)
2	1 (8)	1 (6)	0	2 (6)
≧ 3	0	3 (19)	0	3 (9)
Local therapy for distant metastasis	5 (42)	0	1 (25)	6 (19)
Surgery for brain	1 (8)	0	0	1 (3)
Radiation therapy for bone	2 (17)	0	1 (25)	3 (9)
Radiation therapy for muscle	1 (8)	0	0	1 (3)
Surgery plus radiotherapy for brain	1 (8)	0	0	1 (3)
Posttreatment clinical stage (yc stage)				
I	6 (50)	10 (63)	0	16 (50)
II	2 (17)	2 (13)	3 (75)	7 (22)
IIIA	3 (25)	1 (6)	0	4 (13)
IIIB	1 (8)	2 (13)	1 (25)	4 (13)
IV	0	1 (6)	0	1 (3)

Values are *n* (%) or median (range). *Cases in which multiple metastases were found simultaneously are also included. *TKI* tyrosine kinase inhibitor, *ICI* immune checkpoint inhibitor, *ECOG* Eastern Cooperative Oncology Group, *COPD* chronic obstructive pulmonary disease, *CEA* carcinoembryonic antigen

### Surgical parameters and pathological features

The surgical parameters and pathological features are presented in Table 2. The indications for salvage surgery were local progression after drug therapy, tumor remission to be resectable after drug therapy, and discontinuation of prior drug therapy in 15 (47%), 16 (50%), and one (3%) patients, respectively. The surgical procedures were lobectomy in 28 patients (88 %), segmentectomy in 2 (6%), and wedge resection in 2 (6%). Twenty-five patients (78%) underwent mediastinal lymph node dissection. The median blood loss volume was 100 mL (range, 0**–**1006 mL), and the median duration of operation was 218 min (range, 47**–**444 min). The measured blood loss volume in the ICI groups was 505, 687, 1006, and 0 mL. Although statistical tests were not performed due to the small number of cases in the ICI group, there was a trend toward major blood loss in the ICI group. A summary of the blood loss data is provided in Additional file 1. Complete resection of the primary tumor was accomplished in 28 (88%) patients. Postoperative complications occurred in six (19%) patients (pulmonary fistula, *n* = 1; arrhythmia, *n* = 2; chylothorax, *n* = 1; and pneumonia, *n* = 2) including a CTCAE grade 3 complication (pneumonia). According to prior therapy, postoperative complications were found in two of 12 patients (17%) in the cytotoxic chemotherapy group, three of 16 patients (19%) in the TKI, and one of four patients (25%) in the ICI group. There was no statistical difference in the complication rates among the groups (*p* = 1.0). While no 30-day surgery-related deaths were observed, one (3%) patient died on the 34th postoperative day due to acute exacerbation of idiopathic interstitial pneumonia, which happened on the fourth postoperative day.

**Table 2:** Surgical parameters and pathological features

Characteristics	Cytotoxic agent (*n* = 12)	TKI (*n* = 16)	ICI (*n* = 4)	All drugs (*n* = 32)
Indications for salvage surgery				
Local progression after drug therapy	3 (25)	11 (69)	1 (25)	15 (47)
Tumor remission to be resectable	8 (67)	5 (31)	3 (75)	16 (50)
Discontinuation of prior drug therapy	1 (8)	0	0	1 (3)
Surgical procedure				
Lobectomy	11 (92)	14 (88)	3 (75)	28 (88)
Segmentectomy	0	1 (6)	1 (25)	2 (6)
Wedge resection	1 (8)	1 (6)	0	2 (6)
Surgical approach				
Video-assisted thoracic surgery	7 (58)	12 (75)	1 (25)	20 (62)
Open chest surgery	5 (42)	4 (25)	3 (75)	12 (38)
Mediastinal lymph node dissection (+)	8 (67)	13 (81)	4 (100)	25 (78)
Bronchial stump coverage	4 (33)	5 (31)	1 (25)	10 (31)
Bronco-/pulmonary artery plasty	0	3 (19)	0	3 (9)
Complete resection of the primary lesion	12 (100)	13 (81)	3 (75)	28 (88)
Median blood loss volume, mL (range)	85 (0–1005)	95 (0–300)	596 (0–1006)	100 (0–1006)
Median duration of operation, min (range)	194 (47–444)	218 (77–293)	345 (182–438)	218 (47–444)
Median duration of a chest drain placement, days (range)	2 (0–5)	3 (2–27)	4 (3–6)	3 (0–27)
Median postoperative hospital stay, days (range)	8 (3–34)	7 (4–30)	13 (5–22)	8 (3–34)
Postoperative complication	2 (17)	3 (19)	1 (25)	6 (19)
Pathological stage (yp stage)				
0 (no residual tumor)	1 (8)	1 (6)	2 (50)	4 (13)
I	4 (33)	8 (50)	0	12 (38)
II	1 (8)	1 (6)	0	2 (6)
IIIA	5 (42)	3 (19)	1 (25)	9 (28)
IIIB	1 (8)	0	0	1 (3)
IV	0	3 (19)	1 (25)	4 (13)
Histologic type				
Adenocarcinoma	9 (75)	16 (100)	3 (75)	28 (88)
Squamous cell carcinoma	2 (17)	0	0	2 (6)
Large cell neuroendocrine carcinoma	1 (8)	0	0	1 (3)
Non-small cell carcinoma (NOS)	0	0	1 (25)	1 (3)
Driver gene alteration				
ALK	0	1 (6)	0	1 (3)
EGFR				
Exon 19 deletion	1 (8)	13 (81)	0	14 (44)
Exon 21 L858R	1 (8)	2 (13)	0	3 (9)
PD-L1 expression				
< 1%	1 (8)	8 (50)	1 (25)	10 (31)
1–50%	1 (8)	0	1 (25)	2 (6)
> 50%	1 (8)	0	2 (50)	3 (9)
unknown	9 (79)	8 (50)	0	17 (53)
Therapeutic effect of prior treatment (Ef)				
Ef. 0	1 (8)	2 (13)	0	3 (9)
Ef. 1	8 (67)	11 (69)	1 (25)	20 (62)
Ef. 2	2 (17)	2 (13)	1 (25)	5 (16)
Ef. 3	1 (8)	1 (6)	2 (50)	4 (13)

Values are *n* (%) or median (range). *TKI* tyrosine kinase inhibitor, *ICI* immune checkpoint inhibitor, *NOS* not otherwise specified, *ALK* anaplastic lymphoma kinase, *EGFR* epidermal growth factor receptor, *PD-L1* programmed death ligand 1, *Ef* pathological therapeutic effect

Regarding the pathological features (Table 2), the pathological stage after therapy (yp stage) was I in 12 (38%), II in two (6%), IIIA in nine (28%), IIIB in one (3%), and IV in four (13%) patients. Pathological complete response (absence of residual tumor) was observed in four (13%) patients, among which two (50%) patients were in the ICI group. The histological type was adenocarcinoma in 28 (88%) patients, squamous cell carcinoma in two (6%) patients, and others in two (6%) patients. Regarding driver gene alteration, EGFR mutation was positive in 17 (53%) patients (exon 19 deletion, *n* = 14 and exon 21 L858R, *n* = 3), and ALK rearrangement was found in one (3%) patient. In the TKI group, EGFR T790M mutation was found in seven of 16 (44%) patients at the time of salvage surgery. Programmed death-ligand 1 (PD-L1) expression in the primary tumor was evaluated in 15 (46%) patients; five (15%) patients showed PD-L1 expression (1–50% in two [6%] and > 50% in three [9%]). The pathological treatment effect was determined as Ef. 0 in three patients (9%), Ef. 1 in 20 (62%), Ef. 2 in five (16%), and Ef. 3 in four (13%).

### Clinical course after salvage surgery

Details of the clinical course after salvage surgery, including postoperative drug treatment, postoperative recurrence, and treatment for recurrence, are shown in Table 3. Postoperative treatment was performed in 21 (66%) patients (cytotoxic chemotherapy, *n* = 7 [22%]; CRT/RT, *n* = 3 [9%]; TKI therapy, *n* = 8 [25%; including a patient in the cytotoxic agent group], and RT to the mediastinum, *n* = 3 [9%]). Over a median postoperative follow-up duration of 27 months (range, 2**–**131 months), postoperative recurrence was observed in 23 patients (72%); locoregional and distant recurrences occurred in four (13%) and 19 (59%) patients, respectively. The number of recurrences after salvage surgery according to the type of prior drug therapy was 8 (67%) in the cytotoxic agent group, 13 (81%) in the TKI group, and two (50%) in the ICI group. Regarding the treatment for recurrence after salvage surgery, the cytotoxic agent was administered to six (19%) patients, ICI plus cytotoxic agent to one (3%) patient, ICI alone to two (6%) patients, TKI alone to six (19%) patients, and TKI followed by the cytotoxic agent to three (9%) patients. Among 13 patients with recurrence in the TKI group, seven continued to receive TKI therapy (reuse of initial TKI in three patients and switching first- or second- to third-generation TKI in four patients). Two patients in the cytotoxic agent group and no patients in the ICI group received TKI therapy for recurrence after surgery. Regarding postoperative treatment in the TKI group, seven of 16 (44%) patients continued TKI therapy using the same or another TKI. Among these seven patients, four experienced re-recurrence 2, 7, 10, and 24 months after surgery, and they survived with the disease for 25, 79, 27, and 37 months after surgery. On the other hand, the other three patients survived without re-recurrence during the follow-up period of 10–60 months after surgery.

**Table 3:** Clinical course after salvage surgery

Characteristics	Cytotoxic agent (*n* = 12)	TKI (*n* = 16)	ICI (*n* = 4)	All drugs (*n* = 32)
Postoperative treatment				
Cytotoxic chemotherapy	5 (42)	1 (6)	1 (25)	7 (22)
Chemoradiotherapy/radiation therapy	0	3 (19)	0	3 (9)
TKI therapy	1 (8)	7 (44)	0	8 (25)
Radiation therapy to the mediastinum	2 (17)	1 (6)	0	3 (9)
Recurrence after salvage surgery	8 (67)	13 (81)	2 (50)	23 (72)
Initial metastatic/progressive site after surgery				
Locoregional	0	4 (32)	0	4 (13)
Distant	8 (67)	9 (56)	2 (50)	19 (59)
Treatment for recurrence after salvage surgery				
Systemic treatment				
Cytotoxic chemotherapy	2 (17)	2 (13)	2 (50)	6 (19)
Cytotoxic chemotherapy plus ICI therapy	1 (8)	0	0	1 (3)
ICI therapy	2 (17)	0	0	2 (6)
TKI therapy	1 (8)	5 (31)	0	6 (19)
TKI plus cytotoxic chemotherapy	1 (8)	2 (13)	0	3 (9)
Local treatment				
Radiation therapy	4 (25)	7 (44)	0	11 (34)
Surgery for metastatic site	2 (17)	0	0	2 (6)

Values are *n* (%). *TKI* tyrosine kinase inhibitor, *ICI* immune checkpoint inhibitor. Values are *n* (%) or median (range)

### Prognosis and prognostic factors after salvage surgery

**Fig. 1:**
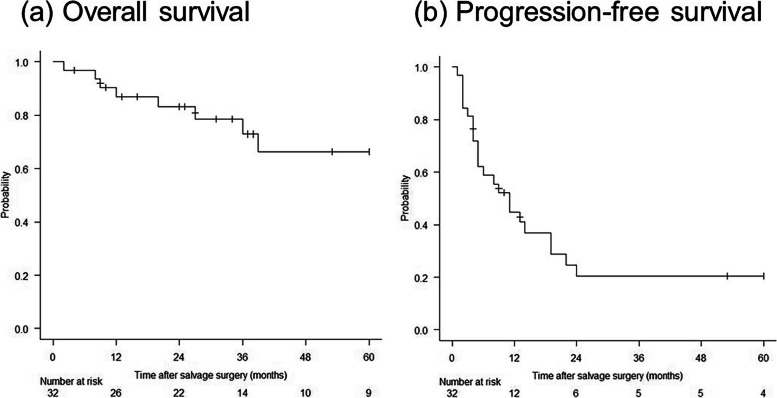
Survival curves of patients after salvage surgery. **a** OS. **b** PFS. The 2- and 5-year OS rates are 83% and 66%, respectively, while the 2- and 5-year PFS rates are 21%. OS, overall survival; PFS, progression-free survival

**Fig. 2:**
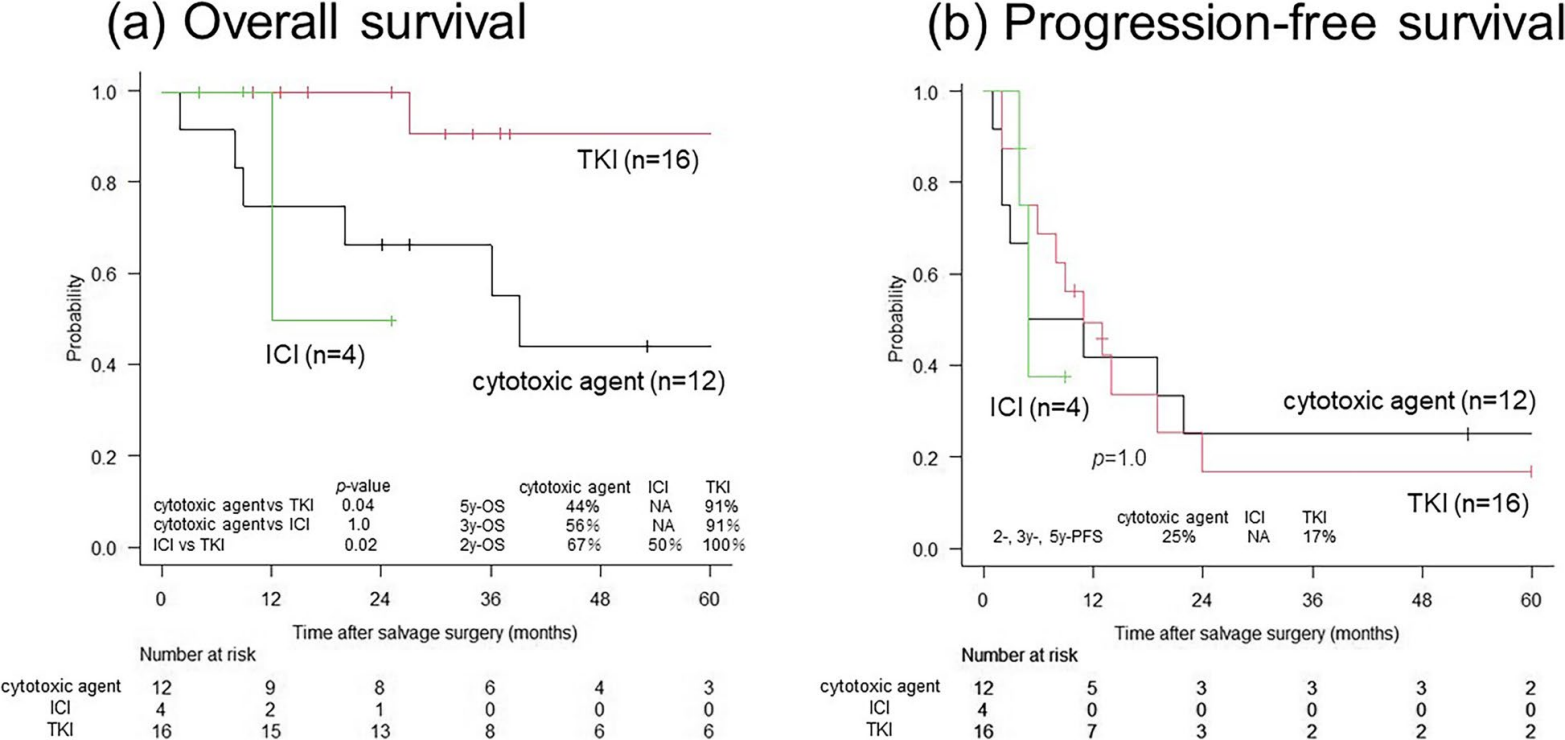
Survival curves of patients after salvage surgery according to the types of prior drug therapy. **a** OS. **b** PFS. The 2-year OS rates are 67%, 100%, and 50% in the cytotoxic agent, TKI, and ICI groups, respectively. The 2-year PFS rates are 25%, 17%, and not assessable in the cytotoxic agent, TKI, and ICI groups, respectively. There are significant differences in OS between the TKI and cytotoxic agent groups (*p* = 0.04) and the TKI and ICI groups (*p* = 0.02). There is no significant difference in PFS among the three groups (*p* = 1.0). ICI, immune checkpoint inhibitor; NA, not available; OS, overall survival; PFS, progression-free survival; TKI, tyrosine kinase inhibitor

Figure 1 shows Kaplan**-**Meier curves of OS and PFS after surgery. The2-, 3-, and 5-year OS rates were 83%, 73%, and 66%, respectively. The 2-, 3-, and 5-year PFS rates were all 21%. The survival curves according to the type of prior drug therapy are shown in Fig. 2. The 2-year OS rates for the cytotoxic agent, TKI, and ICI groups were 67%, 100%, and 50%, respectively. Meanwhile, the 2-year PFS rates for these groups were 25%, 17%, and unavailable, respectively. Despite the small size of each group, there was a significant difference in OS between the TKI and cytotoxic agent groups (*p* = 0.04), as well as between the TKI and ICI groups (*p* = 0.02). There was no significant difference in PFS among the groups. Table 4 shows the predictive factors for OS and PFS as determined by univariate and multivariate analyses. In univariate analysis, prior treatment using TKI (as compared to cytotoxic chemotherapy or ICI) and preoperative *CEA* < 5 ng/mL (as compared to ≥ 5 ng/mL) were significant predictors for OS, while yp stages I**–**III (as compared to yp stage IV) and preoperative *CEA* < 5 ng/mL (as compared to ≥ 5 ng/mL) were significant for PFS. Variables with *p* < 0.15 in univariate analysis were prior TKI therapy, preoperative CEA, and histology for OS and age, yp stage, initial CEA, and preoperative CEA for PFS, and these factors were included in multivariate analysis. In multivariate analyses, prior TKI therapy (hazard ratio [HR] 0.06, 95% confidence interval [CI] 0.006**–**0.68) and preoperative *CEA* < 5 ng/mL (*HR* 0.03, 95% *CI* 0.002**–**0.41) were independent prognostic factors for OS. However, no independent prognostic factors for PFS were identified.

**Table 4:** Univariate and multivariate analyses for overall and progression-free survival

Variables (favorable factor)	OS			PFS		
	Univariate	Multivariate		Univariate	Multivariate	
	*p*	HR (95% *CI*)	*p*	*p*	HR (95% *CI*)	*p*
Age (under 75)	0.99	-	-	0.06	-	-
Sex (female)	0.99	-	-	0.47	-	-
c stage (stage III [vs. IV])	0.62	-	-	0.80	-	-
yc stage (stages I–III [vs. IV])	0.61	-	-	0.97	-	-
yp stage (stages I–III [vs. IV])	0.19	-	-	0.03	0.40 (0.12–1.32)	0.13
Prior treatment (TKI)	0.04	0.06 (0.006–0.68)	0.02	0.86	-	-
Initial serum CEA level (under 5 ng/mL)	0.39	-	-	0.10	-	-
Preoperative serum CEA level (under 5 ng/mL)	0.007	0.03 (0.002–0.41)	0.01	0.04	0.49 (0.21–1.17)	0.11
Surgical mode (lobectomy)	0.99	-	-	0.25	-	-
Surgical mode (segmentectomy or more)	0.99	-	-	0.40	-	-
Mediastinal lymph node dissection (yes)	0.73	-	-	0.73	-	-
Histology (non-adenocarcinoma)	0.11	-	-	0.82	-	-
Pathological complete response of primary lesion (yes)	0.99	-	-	0.99	-	-
Postoperative treatment (yes)	0.30	-	-	0.58	-	-

*OS* overall survival, *PFS* progression-free survival, *HR* hazard ratio, *CI* confidence interval, *TKI* tyrosine kinase inhibitor, *CEA* carcinoembryonic antigen

**Fig. 3:**
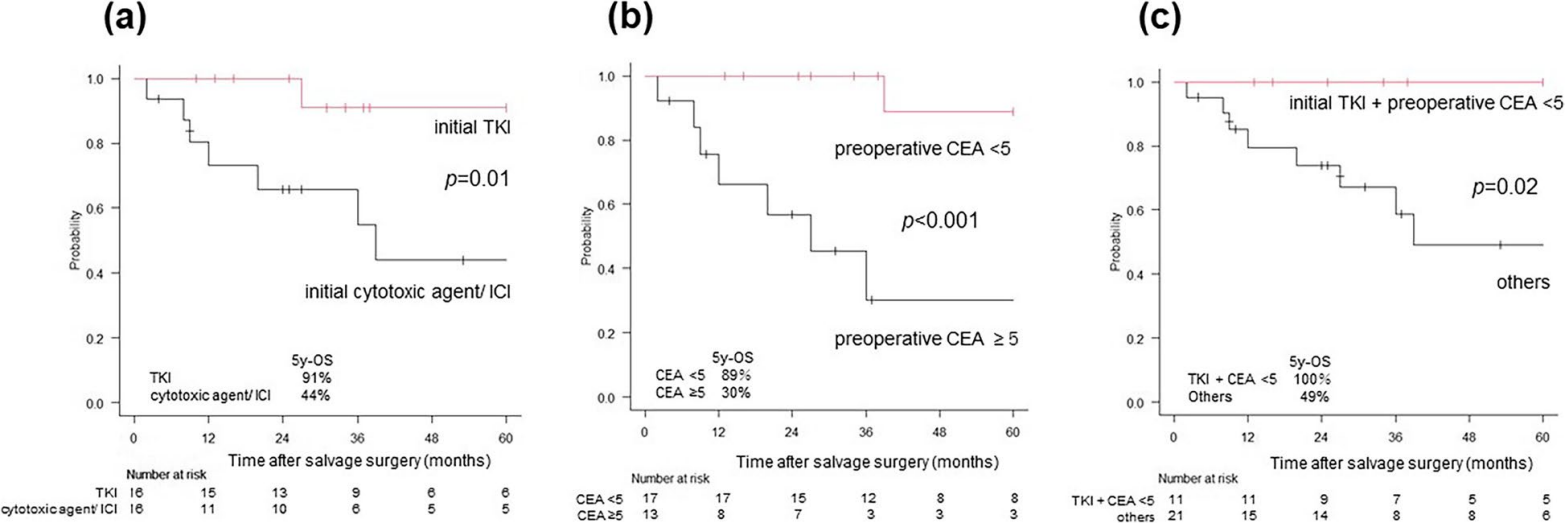
OS curves in patients after salvage surgery. The patients were stratified by prior drug treatment (TKI or others) and preoperative CEA level (*CEA* < 5 or ≥ 5 ng/mL). **a** OS curves stratified by prior drug treatment. The 5-year OS rates are 91% and 44% in the TKI and cytotoxic agents or ICI groups, respectively. There is a significant difference between them (*p* = 0.01). **b** OS curves stratified by preoperative CEA level. The 5-year OS rates are 89% and 30% in the *CEA* < 5 and *CEA* ≥ 5 ng/mL groups, respectively. There is a significant difference between them (*p* < 0.001). **c** OS curves stratified by the two prognosticators of OS (prior drug treatment and preoperative CEA level). The 5-year OS rates are 100% and 49% in the prior TKI therapy plus *CEA* < 5 ng/mL group and the others, respectively. There is a significant difference between them (*p* = 0.02). CEA, carcinoembryonic antigen; ICI, immune checkpoint inhibitor; OS, overall survival; TKI, tyrosine kinase inhibitor

OS curves stratified by the two prognosticators (prior TKI therapy and preoperative CEA <5 ng/mL) are shown in Fig. 3. When stratified by prior drug therapy (Fig. 3a), a significant difference was found between the TKI group and the cytotoxic agent or ICI group (*p* = 0.01; 5-year OS: 91% vs. 44%). However, when stratified by preoperative CEA (Fig. 3b), a significant difference was found between the preoperative *CEA* < 5 ng/mL and preoperative *CEA* ≥ 5 ng/mL group (*p* < 0.001; 5-year OS: 89% vs. 30%). Moreover, when stratified by the two prognosticators of OS (Fig. 3c), there was a significant difference between the group that met both favorable prognosticators (prior TKI therapy plus preoperative *CEA* < 5 ng/mL) and the group that did not (*p* = 0.02; 5-year OS: 100% vs. 49%).

## Discussion

We showed the safety and efficacy of salvage surgery after systemic drug therapy in patients with advanced-stage NSCLC. Moreover, our study findings suggest that prior TKI therapy and preoperative *CEA* < 5 ng/mL are favorable prognostic factors in patients receiving salvage surgery after drug therapy. Previous literature on salvage surgery after systemic drug therapy [9, 14–19] is summarized in Table 5. The present study has two characteristics compared with previous studies. First, the present study includes patients who underwent salvage surgery after cytotoxic chemotherapy, while there have been a few case reports about such kind of salvage surgery [20, 21]. Second, this study also includes patients undergoing salvage surgery following TKI or ICI therapy, and we can compare the three groups categorized by prior drug therapy.

**Table 5:** Previous literature on salvage surgery after systemic drug therapy in non-small cell lung cancer

No.	Author (year)	*N*	Prior drug therapy	Operative mode	^*1^Blood loss volume (ml)	^*1^Operation time (min)	Morbidity (%)	Mortality (%)	OS	PFS
**1**	Hishida (2010)	9	TKI	L 6, BL 1, P 2	NA	NA	11	90 days 0	3-year OS 50% Median OS 32 m	3-year PFS 25 Median PFS 6 m
**2**	Song (2020)	9	TKI	L 9	120 (100–840)	110 (80–170)	11	90 days 0	Median OS 17 m	NA
**3**	Li (2021)	18	TKI	L 18	NA	NA	≧ G4 0	90 days 0	Median OS not reached	Median PFS after initial TKI 23 m
**4**	Ohtaki (2021)	36	TKI	W 2, S 3, L 28, BL 1, P 2	90 (16–340)	225 (153–267)	25 (≧ G3 6)	90 days 0	3-year OS 75%	3-year PFS 22%
**5**	Bott (2018)	9	ICI	W 3, L 4, BL 2	98 (3–500)	168 (45–394)	42 (≧ G3 12)	90 days 0	NA	
**6**	Bertolaccini (2021)	8	Cytotoxic agent plus ICI	L 7, P 1	NA	179 (122–246)	22 (≧ G3 12)	90 days 0	^*2^NA	
**7**	Smith (2021)	6	ICI (17%), cytotoxic agent plus ICI (83%)	W 1, S 1, L 4	125 (65–300)	125 (10–400)	17 (≧ G3 0)	90 days 0	^*3^NA	
**8**	Present study (2022)	32	Cytotoxic agent (38%), TKI (50%), ICI (3%), cytotoxic agent plus ICI (9%)	W 5, S 2, L 37, P 2	100 (0–1006)	218 (47–444)	19 (≧ G3 11)	30 days 0 90 days 3	5-year OS 66%	5-year PFS 21%

*N* number, *OS* overall survival, *PFS* progression-free survival, *TKI* tyrosine kinase inhibitor, *L* lobectomy, *BL* bilobectomy, *P* pneumonectomy, *NA* not available, *G* grade, *W* wedge resection, *S* segmentectomy, *ICI* immune-checkpoint inhibitor. ^*1^Values are described as median (range). ^*2^Six patients were alive without recurrence, while two patients died due to recurrence during the follow-up period (median 15 m). ^*3^All patients remain disease-free during the follow-up period (5–22 m)

In terms of safety, the postoperative complication rate was 19%, while the 90-day mortality rate was 3%. The postoperative complication rates according to the prior drug therapy were 17% in the cytotoxic agent group, 19% in the TKI group, and 25% in the ICI group. There was no statistical difference in the complication rates among the groups (*p* = 1.0). The postoperative complication rates in each drug therapy group are consistent with the safety data from previous studies [9, 14–19]. In previous studies, postoperative complication rates in salvage surgery after TKI [9, 14–16] and ICI plus chemotherapy [17–19] were 11**–**25% and 17**–**42%, respectively. No 90-day mortality was reported in these studies. The results of present and previous studies show that the safety of salvage surgery after systemic drug therapy is considered acceptable. As shown in Table 2 and Additional file 1, there is a possibility that intraoperative blood loss is higher in the ICI group, but we should re-examine this result with a sufficient number of patients in each group to confirm this in the future.

Regarding the treatment effectiveness, this study showed 5-year OS and PFS rates after salvage surgery of 66% and 21%, respectively. Although there was no significant difference in PFS among the groups according to the type of prior drug therapy, a significant difference was found in OS between the TKI group and the other two groups. These results show that patients in the TKI group survived longer with cancer than patients in the other groups. This could be because patients in the TKI group have more treatment options for persistent or recurrent diseases such as reusing initial TKI, which is made possible by resecting TKI-resistant tumors or switching to other TKIs. In the present study, seven of 16 (44%) patients in the TKI group continued TKI therapy using the same or another TKI. Among these seven patients, four experienced re-recurrence 2, 7, 10, and 24 months after surgery, and they survived with the disease for 25, 79, 27, and 37 months after surgery.

In the previous literature, Hishida et al. reported long-term survival outcomes of four patients after salvage surgery following TKI therapy. During a median follow-up period of 65 (48–94) months, three of four patients were alive with the disease, and the one patient receiving TKI therapy was alive without the disease [14]. In addition, Ohtaki et al. reported on the survival of 36 patients who underwent salvage surgery after TKI therapy; the 3-year OS and recurrence-free survival rates after surgery were 75% and 22%, respectively [9]. They also evaluated patients’ data with long-term outcomes after postoperative recurrence by TKI therapy. Sixteen of 28 (57%) recurrent patients survived for > 3 years, among which five (18%) patients survived for > 5 years [9]. In our 16 patients in the TKI group, the 2-year OS rate was 100%, while the 2-year PFS rate was 17%. These data were compatible with those of previous studies showing good OS rates with the disease after salvage surgery following TKI therapy [9, 14]. Although postoperative TKI therapy has been reported to extend PFS after surgery in patients with completely resected EGFR-mutant NSCLC [22–24], there is little literature on the effectiveness of postoperative TKI therapy for patients undergoing salvage surgery for NSCLC. Further studies should be required to judge the effectiveness of postoperative TKI therapy after salvage surgery.

Herein, we discuss the meaning of adding surgery after drug therapy for systemic diseases. Some retrospective studies [25–27] and the randomized phase 2 trial (NCT01725165) [28, 29] have shown the benefits of adding local consolidative therapy (LCT; surgery or RT) after prior drug therapy for oligometastatic NSCLC. Gomez et al. explained that LCT would help remove the burden of treatment-resistant cells that could cause tumors to spread after treatment [28, 29]. Although the best LCT for oligometastatic NSCLC is unclear, surgery can be the first choice with the advantage of histological evaluation. Especially when local progression occurs after TKI therapy, tumor mutation status should be reassessed to select appropriate treatment. Ohtaki et al. reported that six patients switched from first- to third-generation TKI after salvage surgery, resulting in five patients (83%) sustaining disease control for > 18 months [9]. Furthermore, Li et al. reported the benefit of adding surgery when the tumor shrank from prior TKI therapy in stages IIIB**–**IV NSCLC [16]. They compared 73 patients who received TKI therapy to 18 who underwent salvage surgery after TKI therapy and reported a significantly longer PFS in the surgery group (23.4 months vs. 12.9 months, *p* < 0.001). Half of the patients in the surgery group experienced distant metastasis without locoregional recurrence during the median follow-up period of 27.5 months. These results show that salvage surgery cannot control the systemic disease condition but can prolong PFS through local disease control [16]. Based on these reports [9, 16, 28, 29], we considered that some patients would benefit from surgery after drug therapy, even in systemic diseases.

Regarding the appropriate indications for salvage surgery after systemic drug therapy, we demonstrated that “prior TKI therapy” and “preoperative CEA < 5 ng/mL” were independent prognosticators for better OS. This result is in agreement with that reported by Ohtaki et al., who showed that preoperative CEA (≥ 5 ng/mL) was a bad independent prognosticator of OS (*HR* 4.84, *p* = 0.005) [9]. We speculated that a high CEA level might represent a higher tumor burden and widespread micrometastasis, resulting in poor OS. In general, CEA was known as a predictive marker for the risk of recurrence and risk of death in NSCLC patients [30]. Moreover, changes in perioperative CEA levels were shown to be prognostically beneficial. More than 65% of patients with high preoperative CEA levels had normalized CEA levels after curative resection, and the prognosis for them was better than for those with high postoperative CEA levels [31]. Patients with prior TKI therapy have more treatment options for persistent or recurrent diseases, resulting in better OS. Therefore, we suggest that “prior TKI therapy” and “preoperative CEA < 5 ng/mL” are important indicators to select appropriate candidates for salvage surgery after drug therapy.

### Limitation

There are several limitations in this study. Firstly, despite being a multi-institutional study aimed at collecting as many study participants as possible, the current retrospective investigation only included a small number of highly selected cases. Additionally, since this study is retrospective, there might have been differences in the policy for salvage surgery among the facilities. To accurately assess the real effectiveness of salvage surgery for NSCLC, future studies need to be prospective with strictly defined inclusion criteria and should include nonsurgical cases as well. Second, the use of three types of drug therapy overlapped in the same patient before or after surgery. However, sequential or concurrent use of different types of drug therapy is common in clinical practice. Therefore, it was difficult to avoid overlapping medications in this retrospective study.

## Conclusion

Prior TKI therapy and preoperative CEA < 5 ng/mL were favorable prognostic factors for OS in patients with NSCLC treated with salvage surgery after drug therapy. Patients who meet both favorable prognostic factors are considered good candidates for salvage surgery after drug therapy.

### Supplementary Information

**Additional file 1. **Blood loss volume in salvage surgery according to the types of prior drug therapy. ICI: Immune checkpoint inhibitor; TKI: Tyrosine kinase inhibitor. 

## Data Availability

Research data supporting this publication is available upon editor’s request.

## Abbreviations

ALK: Anaplastic lymphoma kinase
CEA: Carcinoembryonic antigen
CI: Confidence interval
CRT: Chemoradiotherapy
CT: Computed tomography
CTCAE: Common Terminology Criteria for Adverse Events
Ef: Pathological treatment effect
EGFR: Epidermal growth factor receptor
HR: Hazard ratio
ICI: Immune checkpoint inhibitor
LCT: Local consolidative therapy
NSCLC: Non-small cell lung cancer
OS: Overall survival
PFS: Progression-free survival
PD-L1: Programmed death-ligand 1
PS: Performance status
TKI: Tyrosine kinase inhibitor
