# Supplementary material for: Prognostic factors affecting survival in patients with non-small cell lung cancer treated with salvage surgery after drug therapy: a multi-institutional retrospective study
Source: World J Surg Oncol. 2023 Sep 15;21:290. doi: 10.1186/s12957-023-03177-5 (PMC10503184; doi:10.1186/s12957-023-03177-5)
Supplement: Supplementary file 1 — Additional file 1. Blood loss volume in salvage surgery according to the types of prior drug therapy. ICI: Immune checkpoint inhibitor; TKI: Tyrosine kinase inhibitor. [file 12957_2023_3177_MOESM1_ESM.pptx]

## Slide 1
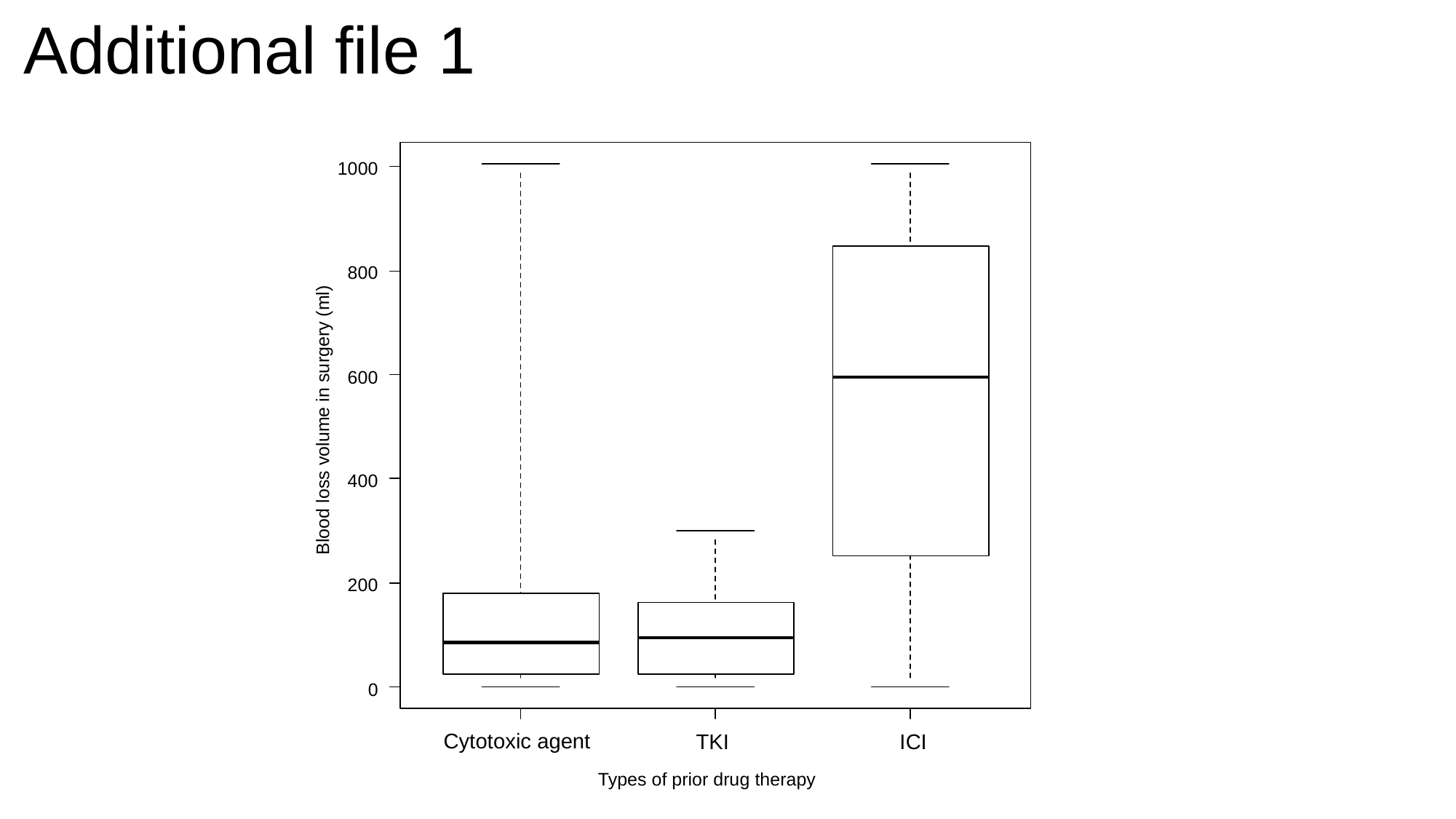

# Additional file 1
Blood loss volume in surgery (ml)
Cytotoxic agent
ICI
TKI
Types of prior drug therapy
